# *Methylobacterium* Genome Sequences: A Reference Blueprint to Investigate Microbial Metabolism of C1 Compounds from Natural and Industrial Sources

**DOI:** 10.1371/journal.pone.0005584

**Published:** 2009-05-18

**Authors:** Stéphane Vuilleumier, Ludmila Chistoserdova, Ming-Chun Lee, Françoise Bringel, Aurélie Lajus, Yang Zhou, Benjamin Gourion, Valérie Barbe, Jean Chang, Stéphane Cruveiller, Carole Dossat, Will Gillett, Christelle Gruffaz, Eric Haugen, Edith Hourcade, Ruth Levy, Sophie Mangenot, Emilie Muller, Thierry Nadalig, Marco Pagni, Christian Penny, Rémi Peyraud, David G. Robinson, David Roche, Zoé Rouy, Channakhone Saenampechek, Grégory Salvignol, David Vallenet, Zaining Wu, Christopher J. Marx, Julia A. Vorholt, Maynard V. Olson, Rajinder Kaul, Jean Weissenbach, Claudine Médigue, Mary E. Lidstrom

**Affiliations:** 1 Département Micro-organismes, Génomes, Environnement, Université de Strasbourg, UMR7156 CNRS, Strasbourg, France; 2 Department of Chemical Engineering, University of Washington, Seattle, Washington, United States of America; 3 Department of Organismic and Evolutionary Biology, Harvard University, Cambridge, Massachusetts, United States of America; 4 Institut de Génomique, Laboratoire de Génomique Comparative, CEA and UMR8030 CNRS, Génoscope, Evry, France; 5 Genome Center, University of Washington, Seattle, Washington, United States of America; 6 Institute of Microbiology, ETH Zurich, Zurich, Switzerland; 7 Institut de Génomique, CEA, Génoscope, Evry, France; 8 Swiss Institute of Bioinformatics, Vital-IT group, Lausanne, Switzerland; 9 Department of Genome Sciences, University of Washington, Seattle, Washington, United States of America; 10 Department of Medicine, University of Washington, Seattle, Washington, United States of America; 11 Department of Microbiology, University of Washington, Seattle, Washington, United States of America; University of Hyderabad, India

## Abstract

**Background:**

Methylotrophy describes the ability of organisms to grow on reduced organic compounds without carbon-carbon bonds. The genomes of two pink-pigmented facultative methylotrophic bacteria of the Alpha-proteobacterial genus *Methylobacterium*, the reference species *Methylobacterium extorquens* strain AM1 and the dichloromethane-degrading strain DM4, were compared.

**Methodology/Principal Findings:**

The 6.88 Mb genome of strain AM1 comprises a 5.51 Mb chromosome, a 1.26 Mb megaplasmid and three plasmids, while the 6.12 Mb genome of strain DM4 features a 5.94 Mb chromosome and two plasmids. The chromosomes are highly syntenic and share a large majority of genes, while plasmids are mostly strain-specific, with the exception of a 130 kb region of the strain AM1 megaplasmid which is syntenic to a chromosomal region of strain DM4. Both genomes contain large sets of insertion elements, many of them strain-specific, suggesting an important potential for genomic plasticity. Most of the genomic determinants associated with methylotrophy are nearly identical, with two exceptions that illustrate the metabolic and genomic versatility of *Methylobacterium*. A 126 kb dichloromethane utilization (*dcm*) gene cluster is essential for the ability of strain DM4 to use DCM as the sole carbon and energy source for growth and is unique to strain DM4. The methylamine utilization (*mau*) gene cluster is only found in strain AM1, indicating that strain DM4 employs an alternative system for growth with methylamine. The *dcm* and *mau* clusters represent two of the chromosomal genomic islands (AM1: 28; DM4: 17) that were defined. The *mau* cluster is flanked by mobile elements, but the *dcm* cluster disrupts a gene annotated as chelatase and for which we propose the name “island integration determinant” (*iid*).

**Conclusion/Significance:**

These two genome sequences provide a platform for intra- and interspecies genomic comparisons in the genus *Methylobacterium*, and for investigations of the adaptive mechanisms which allow bacterial lineages to acquire methylotrophic lifestyles.

## Introduction

Pink-pigmented facultative methylotrophs of the genus *Methylobacterium* are ubiquitous in soil, air and water environments [1]. The common trait of all *Methylobacterium* species is the ability to grow on one or several reduced one carbon (C1) compounds other than methane, most prominently methanol, which is a major volatile organic compound emitted by vegetation [2]. Accordingly, strains of *Methylobacterium* are often found in association with plants, either involved in *bona fide* symbioses as endophytes, or as epiphytes on leaf surfaces [3]–[7]. The potential of strains from this genus to provide biotechnological products of high added value has attracted sustained scientific attention [8], [9].

**Figure 1:**
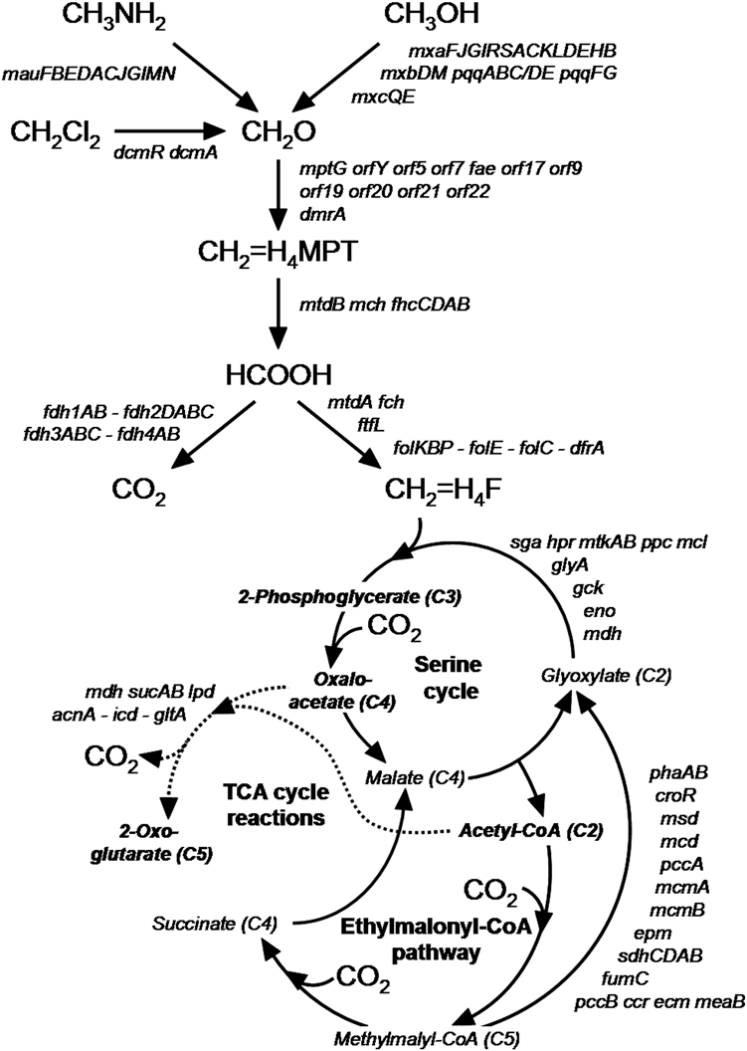
Central pathways for carbon conversion in *Methylobacterium* during methylotrophic growth. Full lines, H_4_MPT-dependent pathway, H_4_F-dependent pathway, serine cycle and ethylmalonyl-CoA pathway for glyoxylate regeneration; broken line, tricarboxylic acid cycle reactions (2-oxoglutarate dehydrogenase activity (and a dissimilatory TCA cycle) are not essential for methylotrophic growth [93], [94]). Key pathway outputs [13] used for carbon assimilation (biomass production) are shown in bold italics. Genes involved in the serine cycle, the TCA cycle and in the ethylmalonyl-CoA pathway are indicated. Genes given on the same line and not separated by hyphens are closely associated on the chromosome. Genes and their arrangement on the chromosome are strongly conserved in strains AM1 and DM4 (see Suppl. Table S1), except the *mau* cluster for methylamine utilization and the *dcm* cluster for dichloromethane utilization which are unique to strain AM1 and strain DM4, respectively.

Of all *Methylobacterium* strains, *M. extorquens* strain AM1 (formerly *Pseudomonas* AM1, *Methylobacterium* sp. AM1) is the best studied, and has served as a model organism for over four decades. It was first isolated in 1960 in Oxford, England, as an airborne contaminant growing on methylamine [10]. It was then used as a workhorse to characterize the serine cycle for assimilation of the C1-unit of methylene tetrahydrofolate, a central intermediate in methylotrophic metabolism, and more recently the ethylmalonyl-CoA pathway for glyoxylate regeneration [8], [11]–[13] (Fig. 1). Enzymatic systems for oxidation of both methanol [14], [15] and methylamine [16], which involve the use of specific cofactors pyrroloquinoline quinone (PQQ) and tryptophan tryptophylquinone (TTQ), respectively [17], were characterized in strain AM1. Bacterial tetrahydromethanopterin (H_4_MPT)-dependent enzymes, now known to occur in most methylotrophs [18], [19] but originally thought to be unique to archaeal methanogens [20] were also first demonstrated in this strain. In *Methylobacterium*, the H_4_MPT-dependent pathway has been shown to play a major role in both energy generation and protecting cells from formaldehyde poisoning [21]. As to the analogous tetrahydrofolate (H_4_F)-linked pathway that involves two enzymes encoded by *mtdA* and *fch*, also first discovered in this organism [22], [23], its major role in assimilatory metabolism was recently identified in supplying C1 units into the serine cycle [24], [25] (Fig. 1).

Draft genome data for *M. extorquens* AM1 have been available since 2003 [11] and have enabled transcriptomic and proteomic approaches (see e.g. [26], [27]), as well as metabolomic studies (see e.g. [24], [28], [29]). Combined with the large complement of genetic tools developed for *Methylobacterium* (see e.g. [30], [31]), this has established *M. extorquens* AM1 as a model for systems level investigations.

*Methylobacterium* strain DM4 has been isolated from industrial wastewater sludge in Switzerland, as part of efforts to characterize microorganisms able to degrade the organohalogenated pollutant dichloromethane (DCM) [32]. Unlike methanol and methylamine, which are mainly produced naturally, DCM is better known as a synthetic compound [33], [34]. Rated as potentially carcinogenic for humans and the most highly produced chlorinated organic compound (http://www.eurochlor.org/solvents), DCM is highly volatile (b.p. 38°C) and water-soluble, making it a widespread contaminant in the environment [35]. Aerobic methylotrophic bacteria capable of using DCM as the sole source of carbon and energy [36] express high levels of DCM dehalogenase, which transforms DCM into formaldehyde and two molecules of HCl [37]. The genotoxic effects of DCM in both mammals [38] and bacteria [39] are due to a short-lived intermediate in the enzymatic transformation of DCM to formaldehyde [40]. Growth with DCM, as the main trait that distinguishes strain DM4 from *M. extorquens* AM1, has led to its classification as a separate *Methylobacterium* species [41]. Basing on 16S rRNA gene sequence and DNA-DNA relatedness, it was recently proposed that strain DM4 should be reclassified as *M. extorquens* [42].

The primary objective of this work was to define a fully assembled and annotated reference genomic blueprint for *Methylobacterium*, to assist future experimental investigations of methylotrophic metabolism by global approaches. We report here complete genomic sequences of strains AM1 and DM4, and describe the genomic make-up and potential for genomic plasticity that underlies the extensive capacity of *Methylobacterium* for physiological adaption to methylotrophic lifestyles. Availability of complete genomic sequences of the two strains also provides the opportunity to define the conserved complements of genes associated with methylotrophy, and to investigate the differences between the two strains associated with strain-specific adaptations.

## Results and Discussion

### Genomic structure

**Figure 2:**
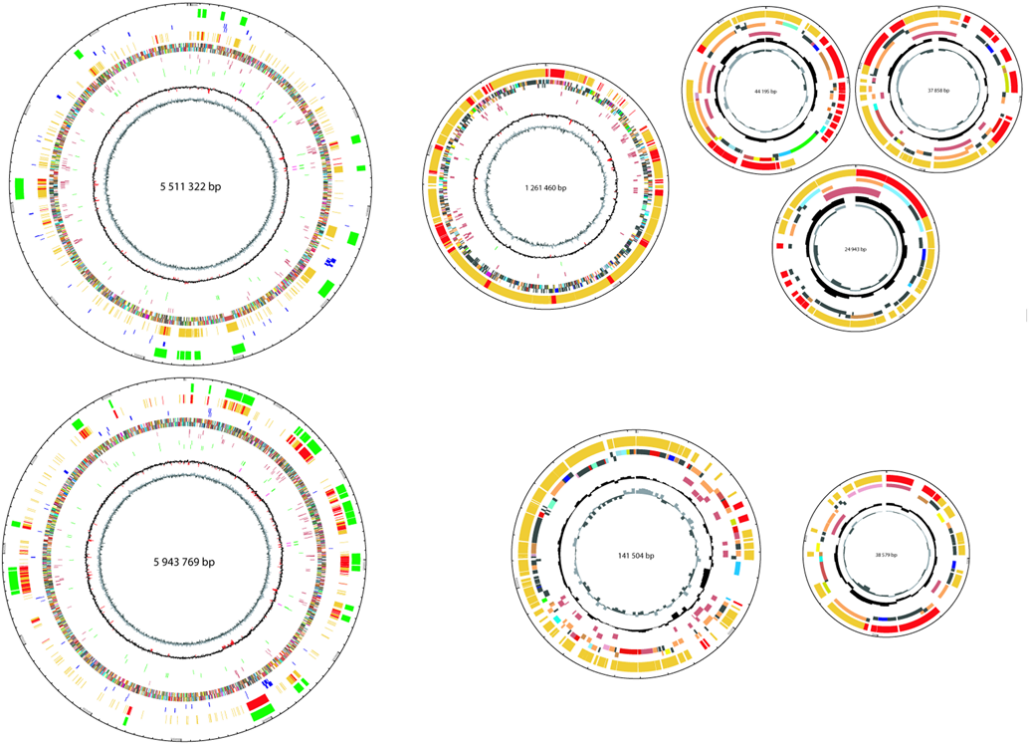
Schematic representation of the 8 circular replicons in the genomes of *Methylobacterium extorquens* strains AM1 (top) and DM4 (bottom). Successive circles from inside to outside: GC skew; GC deviation (with values exceeding +/− 2SD indicated in red); rRNA (pink); tRNA (green); IS elements (brown); all genes coloured according to functional class (COG); methylotrophy genes (blue, see Suppl. Table S1); strain-specific genes (yellow, except genes predicted to be of foreign origin, in red); genomic islands (green, see Table 3). Plasmids are not shown to scale.

The genome of *M. extorquens* AM1 totals 6.88 Mb and consists of five replicons: a chromosome of 5.51 Mbp (Acc. No. CP001510), a megaplasmid of 1.26 Mbp (CP001511), and three plasmids (25 kb (CP001512), 38 kb (CP001513), and 44 kb (CP001514)), with an average GC content of 68.5% (Table 1, Fig. 2). The genome of strain DM4 is somewhat smaller (6.12 Mb), and features only three replicons: a chromosome of 5.94 Mbp (Acc. No. FP103042) and two plasmids (141 kb (FP103043) and 38 kb (FP103044)), with an average GC content of 68.0% (Table 1, Fig. 2). Based on their sizes and the relative distribution of sequencing reads for each replicon, plasmids p1META and p2META are predicted to be present at 2–3 copies per strain AM1 genome, the replicon p3META at 1–2 copies per genome, and the megaplasmid at one copy per genome. Predicted copy numbers of 0.4–0.5 and 0.6–0.7 per genome were obtained for DM4 plasmids p1METDI and p2METDI, respectively.

**Table 1:** Genome statistics for *M. extorquens* AM1 and DM4.

	Strain AM1	Strain AM1	Strain AM1	Strain AM1	Strain AM1	Strain AM1	Strain DM4	Strain DM4	Strain DM4	Strain DM4
	Chromosome	Megaplasmid	Plasmid p1META1	Plasmid p2META1	Plasmid p3META1	Total/average	Chromosome	Plasmid p1METDI	Plasmid p2METDI	Total/Average
Size (bp)	5511322	1261460	44195	37858	24943	**6879778**	5943768	141504	38579	**6123851**
GC (%)	68.7	67.7	67.9	65.3	66.9	**68.5** a	68.1	65.3	63.7	**68.0** a
Repeat regions b (%)	8.3	7.9	0.2	2.7	0.3	**8.0** a	9.3	1.6	0	**9.1** a
Genes	5315	1318	46	45	35	**6759**	5857	137	41	**6035**
Protein-coding genes	5227	1312	46	45	35	**6665**	5769	137	41	**5947**
*Average length*										
CDS (bp)	905.3	846.9	822.7	693.2	536.6	**891.7** a	888.2	878.4	821.9	**887.2** a
Intergenic (bp)	178.1	167.7	163.1	229.2	160.6	**176.4** a	180.4	309.6	202.6	**183.4** a
Coding density (%)	84.2	84.1	78.9	73.1	69.8	**84.0** a	83.6	70.6	77.7	**83.3** a
rRNA operons	5	0	0	0	0	**5**	5	0	0	**5**
tRNA	57	6	0	0	0	**63**	58	0	0	**58**
*Insertion elements (IS)*										
Total length (%)	2.4	7.6	22.0	27.5	15.5	**3.7** a	1.6	21.5	10.0	**2.1** a
Intact IS c	93 (19)	41 (25)	3 (3)	4 (4)	1 (1)	**142 (39)**	54 (27)	15 (15)	2 (2)	**71 (42)**
Partial IS c	8 (7)	22 (13)	1 (1)	1 (1)	0	**32 (19)**	17 (11)	5 (5)	1 (1)	**23 (15)**
MITEs	1	3	0	0	0	4	8	0	0	**8**

a Average.

b Defined by the algorithm Nosferatu as implemented in Mage [45].

c Number of IS elements (number of IS types in brackets).

**Figure 3:**
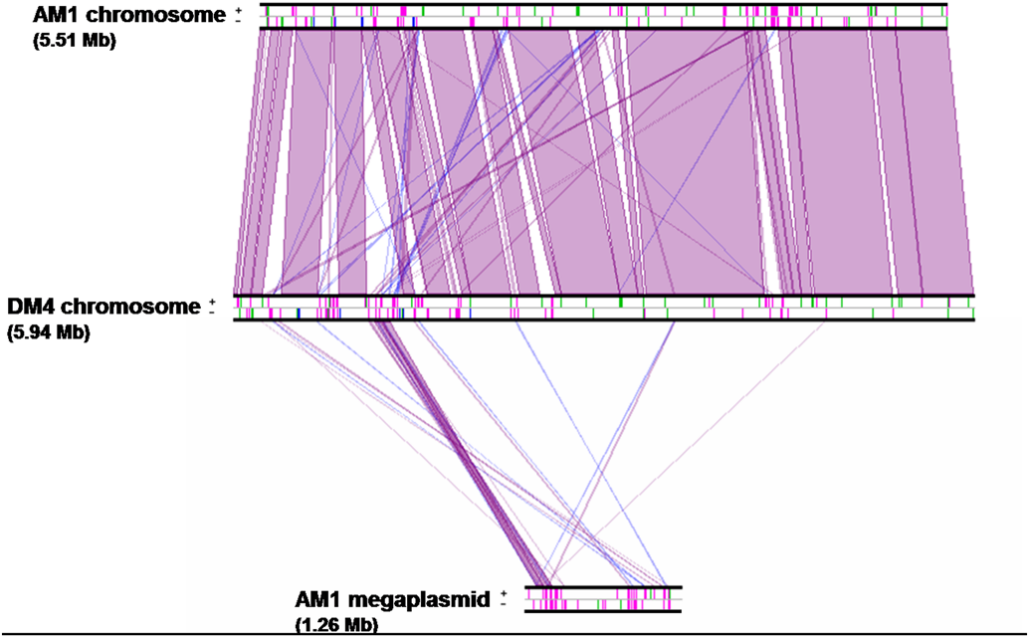
Overall synteny between *M. extorquens* AM1 and DM4. The linearized replicons were aligned and visualized by Lineplot in Mage. Syntenic relationships comprising at least 8 genes are indicated by violet and blue lines for genes found on the same strandor on opposite strands, respectively. IS elements (pink), ribosomal operons (blue) and tRNAs (green) are also indicated.

By convention, the origin of both chromosomes was set upstream of the *dnaA* gene, as no GC skew was observed to help in predict initiation and termination of replication [43]. The chromosomes of the two strains are remarkably similar in both gene content and synteny (Fig. 2, Fig. 3). 85% of the *M. extorquens* AM1 chromosomal genes have full-length homologs (higher than 30% identity) on the chromosome of strain DM4. Of these, 89% have homologs at higher than 95% identity, underlining the orthologous nature of most genes in the two strains. Ribosomal genes (23S, 16S, and 5S) are identical in all five copies of the ribosomal operon of strains AM1 and DM4. The intergenic spacer length between 16S and 23S genes is identical in all five copies of the ribosomal operon of the same strain, but its length differs markedly between the two strains (905 nt in *M. extorquens* AM1, 602 nt in strain DM4). These data confirm the already mentioned recent suggestion [42] that strain DM4 belongs to the species *M. extorquens*.

The distribution of functional categories according to the COG classification ([44], Table 2) was as expected for a free-living proteobacterium with a versatile lifestyle and the observed genome size. COG functional class assignments are more frequent and diverse for chromosomal genes than for plasmid genes (Table 2). No significant differences in functional classes were evident between AM1 and DM4 chromosomes, except for the larger proportion of genes associated with recombination, replication and repair in strain AM1, a reflection of the larger set of IS elements in that strain (Table 1, and see below).

**Table 2:** Functional classes in *M. extorquens* AM1 and DM4 replicons a.

Class - Description	Strain AM1	Strain AM1	Strain AM1	Strain AM1	Strain AM1	Strain AM1	Strain AM1	Strain DM4	Strain DM4	Strain DM4	Strain DM4	Strain DM4
	Chrom.	megaplasmid	p1	p2	p3	Class (%)	Proc. (%) a	chrom.	p1	p2	Class (%)	Proc. (%) a
D - Cell cycle control, cell division, chromosome partitioning	34	7	1	1	1	**0.66**	**16.28**	38	3	2	**0.72**	**17.48**
M - Cell wall/membrane/envelope biogenesis	240	30	1		1	**4.08**		245	4		**4.18**	
N - Cell motility	126	16			3	**2.18**		121	1		**2.05**	
O - Posttranslational modification, protein turnover, chaperones	164	32	2			**2.97**		201	2		**3.41**	
T - Signal transduction mechanisms	266	34	1	1		**4.53**		304	1	1	**5.14**	
U - Intracellular trafficking, secretion, and vesicular transport	35	8	1			**0.66**		32	3		**0.59**	
V - Defense mechanisms	66	13	1			**1.20**		80	2		**1.38**	.
B - Chromatin structure and dynamics	3					**0.05**	**14.12**	4			**0.07**	**13.28**
J - Translation, ribosomal structure and biogenesis	188	14	1			**3.05**		199	1		**3.36**	
K - Transcription	205	46	2	2	2	**3.86**		243	5	2	**4.20**	
L - Replication, recombination and repair	292	161	11	11	3	**7.17**		290	39	7	**5.65**	
C - Energy production and conversion	262	26		1		**4.34**	**24.50**	303	3	2	**5.18**	**27.39**
E - Amino acid transport and metabolism	423	31	1	1		**6.84**		468	12		**8.07**	
F - Nucleotide transport and metabolism	79	11				**1.35**		80			**1.34**	
G - Carbohydrate transport and metabolism	141	9				**2.25**		144	1		**2.44**	
H - Coenzyme transport and metabolism	124	7	1			**1.98**		123	1		**2.08**	
I - Lipid transport and metabolism	164	11				**2.63**		174	2	2	**2.99**	
P - Inorganic ion transport and metabolism	200	35	3	1		**3.59**		220		2	**3.73**	
Q - Secondary metabolites biosynthesis, transport and catabolism	89	12		1		**1.53**		92	1		**1.56**	
R - General function prediction only	355	40	2			**5.96**	**11.24**	388	1	2	**6.57**	**12.12**
S - Function unknown	312	35	2	3		**5.28**		327	2	1	**5.55**	
CDS with at least one COG hit	3768	578	30	22	10	**4408**	**66.15**	4076	84	21	**4181**	**70.27**
Total CDS	5227	1311	46	45	35	**6664**		5772	137	41	**5950**	

a Only the first COG hit of each CDS is considered (CDS may be associated with several COGs).

b Processes: Cellular processes and signaling (D,M,N,O,T,U,V); information storage and processing (B,J,K,L); metabolism (C,E,F,G,H,I,P,Q); and poorly characterized (R,S).

The Mage annotation platform [45] and Alien Hunter [46] were used to detect genes and genome regions found in one strain but not in the other. Several unique chromosomal regions, termed genomic islands and ranging from a few genes to hundreds of genes, which represent approximately 632 kb (11.5%) and 1,054 kb (17.7%) of the chromosome for strains AM1 and DM4, respectively, were defined (Table 3). With the exception of the *dcm* and *mau* gene clusters (see below), few of these islands appear to encode functions important for central metabolism or methylotrophy. One remarkable genomic island in strain AM1 (Table 3) contains a hypothetical gene of unknown function of 47.5 kb (META1_2412) which encodes a 15,831 residue-long repeat-rich polypeptide (Pfam PF00353 (hemolysin-type calcium-binding region); PF05594, (haemagglutinin, bacterial); COG3210 (large exoproteins involved in heme utilization or adhesion), and COG2931 (RTX toxins and related Ca2+−binding proteins). This gene product, if expressed, would represent one of the largest proteins known in biology [47].

**Table 3:** Unique regions in *M. extorquens* AM1 and DM4 chromosomes.

Start CDS (META1_)	Start (nt)	End CDS (META1_)	End (nt)	Length (bp)	[Left border][Inside][Right border] a	IS	Features (proposed role)	Total CDS (%)	Unique CDS b (%)	AH CDS c (%)
*Methylobacterium extorquens* AM1										
0035	37982	0046	51448	13467	[none][GC][IS]	1		14	11 (78.6)	0
tRNA/0058	61782	0073	80559	18778	[tRNA][int-AH][none]	1		17	16 (94.1)	5 (29.4)
0149	156863	tRNA/0165	174721	17859	[none][int-AH-mob][tRNA]	0		16	13 (81.3)	0
tRNA/0241	261958	0273	289656	27699	[tRNA][GC][IS]	4 (1)		34	25 (73.5)	0
1078	1126013	1115	1164815	38802	[IS][GC-mob-tRNA-tRNA][IS]	8		45	19 (42.2)	4 (8.9)
1226	1283284	1257	1308115	24832	[none][GC][tRNA]	0	sulfur metabolism	33	29 (87.9)	0
tRNA/1555	1629314	1614	1691767	62454	[tRNA][none][IS]	2		59	53 (89.8)	0
tRNA/1825	1911338	1924	1995805	84467	[tRNA][GC][int]	6 (1)	copper resistance	100	82 (82.0)	0
2402	2475649	2415/tRNA	2538027	62379	[none][GC][tRNA]	0	giant META1_2412 gene	13	13 (100)	0
2573	2704934	2598	2734385	29452	[none][GC][none]	0	sulfur metabolism	25	14 (56.0)	0
2622	2753794	2652	2781501	27708	[none][GC][none]	0	metal transport	31	31 (100)	0
2657	2787350	META1_2681	2808325	20976	[none][GC][integrase]	0		22	22 (100)	0
2687	2814961	2697	2823774	8814	[none][GC][none]	0		11	10 (90.9)	0
2755	2879429	2817	2939813	60385	[IS][tRNA-AH][tRNA]	2 d	included *mau* gene cluster	63	50 (79.4)	10 (15.9)
tRNA/3934	4056642	4086	4163355	106714	[tRNA][AH][int]	12 (3)	phage-related	148	145 (98.0)	25 (16.9)
4747	4874344	4792/tRNA	4901372	27028	[integrase][GC][tRNA]	5	phage-related	43	41 (95.3)	6 (14.0)
*Methylobacterium extorquens* DM4										
0036	37946	0052	51383	13438	[Mite][int - GC][IS]	2 d		16	13 (81.3)	11 (68.8)
0137	137916	0147/tRNA	146122	8207	[none][AH][tRNA]	2 (1)	SOS repair (*umuCD*)	8	0	4 (50.0)
tRNA/0225	232397	0336	321727	89331	[tRNA][AH][none]	4	sensors/regulators, transport; carbon metabolism	109	62 (56.9)	27 (24.8)
0345	328235	0426	390700	62466	[none][GC][int]	1	Beta-lactamase-like domain repeat region	78	57 (73.1)	12 (15.4)
0707	673123	0725	683063	9941	[int][AH][none]	1	Beta-lactamase-like domain repeat region	17	11 (64.7)	11 (64.7)
0736	689280	0748	702730	13451	[none][AH][IS]	2 (1)	efflux determinant	14	13 (92.9)	13 (92.9)
0769	717385	0782	730568	13184	[none][GC][none]	0	carbon metabolism	13	10 (76.9)	0
0786	733379	0825/tRNA	759448	26070	[none][AH][tRNA]	1 d	gene decay region	39	34 (87.2)	28 (71.8)
tRNA/0840	774084	0935	848713	74630	[tRNA][int-mob(3)-int-AH][none]	8 d (3)	contains putative 5-formyl-H_4_F cyclo-ligase	93	37 (39.8)	30 (32.3)
1157	1065633	1204	1108451	42819	[int][int-int-AH][none]	2 (1)		45	25 (55.6)	19 (42.2)
1209	1114522	1243	1136772	22251	[none][GC][none]	0		34	26 (76.5)	10 (29.4)
1275	1161654	1336	1215594	53941	[none][GC][none]	5 (3)		62	21 (33.9)	19 (30.6)
1341	1219900	1375	1252518	32619	[none][GC-AH][none]	1 d	copper resistance	36	5 (13.9)	3 (8.3)
1382	1257095	1424	1290282	33188	[none][AH][IS]	5	metal resistance	41	35 (85.4)	16 (39.0)
1586	1452662	1650	1512578	59917	[none][GC][none]	7 d (4)		66	54 (81.8)	45 (68.2)
1656	1520241	1683	1548181	27941	[none][GC][none]	0	putative sulfur compound transport/amidase region	28	26 (92.9)	9 (32.1)
1860	1741524	1886	1767524	26001	[none][GC][none]	0	carbon utilisation, transport, molybdopterin-related	25	25 (100)	0
1917	1800884	1956	1830642	29759	[none][GC][none]	3 d	carbon utilisation, transport, amide-related	36	27 (75.0)	18 (50.0)
tRNA/2330	2227106	2333	2244311	17206	[tRNA][none][IS]	1 (1)		4	4 (100)	0
2348	2263918	2380	2300422	36505	[none][GC-AH][none]	0		33	27 (81.8)	6 (18.2)
2551	2460335	2682	2587253	126919	[none][mob-tRNA-int-AH][none]	6 (1)	*dcm* region	129	127 (98.4)	127 (98.4)
tRNA/3361	3293492	3383	3308142	14651	[tRNA][tRNA-int-AH][int]	0		23	19 (82.6)	19 (82.6)
4329	4266669	4356	4283718	17050	[none][AH][int]	3		27	18 (66.7)	10 (37.0)
4367	4291118	4487	4393993	102876	[none][AH][none]	5 d	nitrogen metabolism, urease-like operon	122	108 (88.5)	59 (48.4)
4495	4400484	4514	4415275	14792	[IS][AH][IS]	2		20	16 (80.0)	16 (80.0)
tRNA/4746	4641080	4776	4668490	27411	[tRNA][int-mob-tRNA-AH][none]	0	efflux determinant	30	26 (86.7)	14 (46.7)
tRNA/5356	5288636	5390	5336993	48358	[tRNA][int][none]	0	virulence determinant	35	32 (91.4)	22 (62.9)
tRNA/5552	5522860	5566	5532024	9165	[tRNA][int-AH][none]	20		16	15 (93.8)	15 (93.8)

a int: integrase; mob: mobility determinant; GC: region with atypical GC content; AH: region rich in genes detected by Alien Hunter; IS: Insertion Sequence; MITE: Miniature Inverted Repeat Transposable Element.

b no homolog with >80% identity/0.8 minLrap value in the chromosome of the compared strain.

c as detected by Alien Hunter ([46], see Materials and Methods).

d including one putative MITE.

Extra-chromosomal replicons are highly strain-specific and show little similarity in size, gene content or synteny with each other. However, an approximately 130 kb region of the AM1 megaplasmid is globally syntenic to a region of similar length in the chromosome of strain DM4 (Fig. 3). Plasmids encode mostly proteins of currently unknown function (Table 2) or proteins associated with plasmid-related functions. Exceptions include a cation efflux system on plasmid p1META1 (p1META1_0021/p1META1_0022); a cluster of copper resistance genes on plasmid p2META1 (p2META1_0029/p2META1_0030); a truncated *luxI* gene (p1META1_0049) recently shown to be essential for the operation of two *bona fide*, chromosomally-located *luxI* genes, and encoding two acyl homoserine lactone synthases [48]; and UmuDC systems involved in SOS DNA repair. Unlike in strain DM4, which has two complete copies of *umuDC* on its chromosome (METDI0144/METDI0143 and METDI4328/METDI4329), a complete *umuDC* cluster in AM1 is only found on the megaplasmid (META2_0643/META2_0644), while a truncated copy of *umuC* is found on the chromosome (META1_4790)

### Comparative genomics of aerobic methylotrophy

Methylotrophy can be envisioned in terms of the assembly of discrete metabolic modules, each responsible for a specific metabolic task, which in combination define pathways for methylotrophic metabolism, several variants of which have been well characterized [11], [49].

#### *The* Methylobacterium *blueprint*

In *Methylobacterium*, the currently recognized methylotrophy genes and modules are found exclusively on the chromosomes of strains AM1 and DM4 (Fig. 2, Suppl. Table S1). Common genes associated with methylotrophy inventoried in Suppl. Table S1 display at least 95% identity at the protein level (99.1% average), with complete synteny between the two strains [11]. Several methylotrophy genes are found as singletons, including several cases of genes that encode different subunits of the same enzyme (e.g. *mcmAB*, *pccAB*, see Suppl. Table S1). Nevertheless, a majority of methylotrophy genes are found in large clusters. Only two known methylotrophy gene clusters are not shared between the two strains (Suppl. Table S1, and see below): the *dcm* (dichloromethane degradation) gene region present only in strain DM4, and the *mau* gene cluster encoding methylamine dehydrogenase and accessory functions in strain AM1. One large multi-operon cluster (49.3 kb) encodes most of the serine cycle enzymes, most of the PQQ biosynthesis functions [17], genes for H_4_MPT-linked reactions and H_4_MPT biosynthesis, and H_4_F biosynthesis genes. It also contains genes encoding a homolog of methanol dehydrogenase (XoxFJG) of still unknown function, often found nearby genes involved in C_1_ metabolism [50], [51] and recently suggested to be involved in formaldehyde metabolism in the photosynthetic bacterium *Rhodobacter sphaeroides* [52].

#### Comparison of gene sets for methylotrophy in fully sequenced genomes

A steadily increasing number of genomes of methylotrophic microorganisms have been sequenced, assembled and annotated. We limit our comparative analysis of known genetic determinants and modules of methylotrophy (Suppl. Table S2) to completed, manually annotated and officially published methylotroph genomes (listed in Suppl. Table S3), six of which belong to the phylum *Proteobacteria* and one to the phylum *Verrucomicrobia*. *Methylococcus capsulatus* represents Gamma-proteobacterial methanotrophs [53], while *Methylibium petroleiphilum* [54], *Methylobacillus flagellatus* [55] and *Methylophilales* strain HTCC2181 [56] feature two different orders within Beta-proteobacteria (*Burkholderiales* and *Methylophilales*). *Silicibacter pomeroyi*, although not reported to grow methylotrophically, is an Alpha-proteobacterium of the family *Rhodobacteriaceae* capable of degrading methylated sulfur compounds [57]. *Granulibacter bethesdensis* is an emerging human pathogen of the family of *Acetobacteriaceae* within Alpha-proteobacteria [58] reported to grow on methanol [59]. Finally, strain V4 (candidatus “*Methyloacidiphilum infernorum*”) represents the recently discovered group of thermophilic and acidophilic methanotrophs of the phylum Verrucomicrobia [60].

#### Methanol utilization

The *mxa* gene cluster encoding the classic methanol dehydrogenase is nearly identical (over 99% identity at the protein level) between strains AM1 and DM4, and very similar in the genomes of *M. capsulatus*, *M. flagellatus*, *G. bethesdensis* and several other proteobacterial methylotrophs [61]. This conservation of both gene sequence and gene synteny suggests that the *mxa* gene cluster has most likely disseminated via lateral transfer among methylotrophs of different subclasses of Proteobacteria. This notwithstanding, no similar gene clusters are recognizable in the genomes of the other four organisms discussed here. *M. petroleiphilum* features a gene cluster encoding an alternative methanol dehydrogenase (Mdh2; [61]) with little homology to either *mxaF* or *xoxF*. The gene *xoxF* is found in all of the genomes discussed here except that of *S. pomeroyi* but, as discussed elsewhere [61], the Xox system is unlikely to be responsible for aerobic methanol oxidation. The genes responsible for methanol oxidation by *Methylophilales* HTCC2181 and strain V4 remain unknown, suggesting the existence of other, yet unidentified systems for methanol dissimilation.

#### Methylamine utilization

The *mau* gene cluster encoding the canonical system for methylamine utilization was characterized for a large part in strain AM1, and the genome of *M. flagellatus* [55] contains a *mau* gene cluster very similar to it. The main difference is that the gene for the electron acceptor from methylamine dehydrogenase in strain AM1, amicyanin, is replaced by a gene for azurin, an analogous copper-containing electron acceptor protein in *M. flagellatus*. The *mau* cluster was not found in genomes of the other methylotrophs including strain DM4 discussed here, which were shown or assumed to grow with methylamine (Suppl. Table S2). Thus, as yet uncharacterized genetic determinants are responsible for methylamine utilization in most methylotrophs, including strain DM4 in particular.

#### H_4_MPT-dependent formaldehyde oxidation

Tetrahydomethanopterin (H_4_MPT)-dependent formaldehyde oxidation is the main pathway for both energy generation and formaldehyde detoxification in *M. extorquens*, and therefore absolutely essential for methylotrophy in this organism [21], [62], [63]. First defined in *M. extorquens* AM1, this pathway has also been described in a variety of other bacteria, including from phyla whose methylotrophic ability has not yet been demonstrated such as Planctomycetes [18], [64], [65]. Phylogenetic analysis suggests that this pathway must be one of the most ancient in the context of methylotrophic metabolism. However, it is unessential in *M. flagellatus* [66], and is absent in some other methylotrophs. *S. pomeroyi* possesses an alternative glutathione-dependent (FlhA/FghA) system for oxidation of formaldehyde similar to that of *P. denitrificans* [67] and *R. sphaeroides* [52]. No formaldehyde oxidation systems were identified in the genomes of *Methylophilales* HTCC2181 or *Verrucomicrobia* strain V4.

#### Conversion of formate to CO_2_

*M. extorquens* strains possess four different functional formate dehydrogenases for the final step of energy generation from carbon oxidation [68]. The other methylotrophs included in our analysis also encode one or several FDH homologs (Suppl. Table S2), but only one, FHD2, is consistently detected. These observations suggest that formate oxidation, as a transformation ubiquitous to life, does not strictly qualify as a methylotrophy-specific reaction, and may thus involve analogous [69] enzymatic systems.

#### C1 assimilation via methylene tetrahydrofolate and the serine cycle

**Figure 4:**
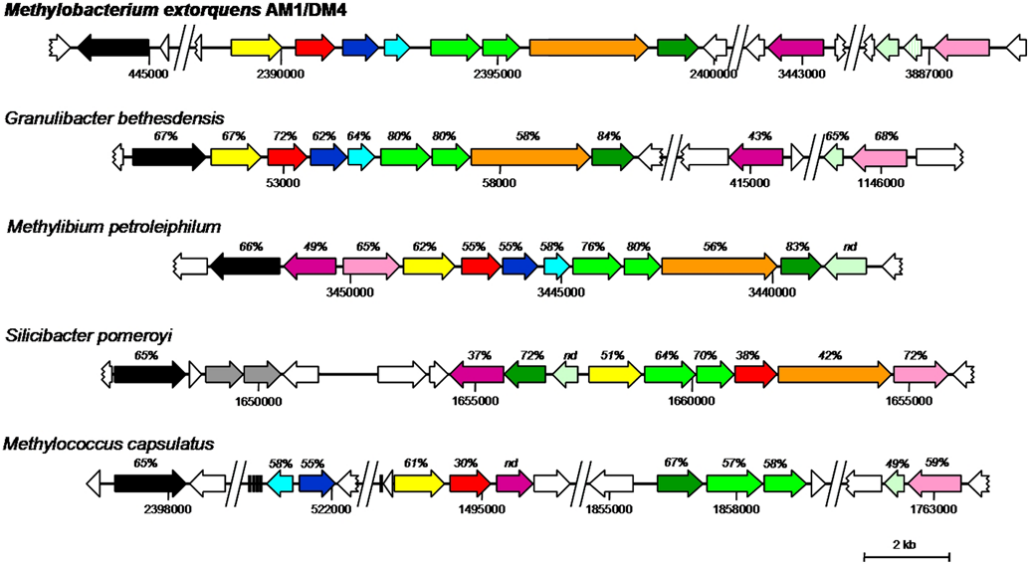
Clustering and conservation of serine cycle and other genes important for methylotrophic metabolism in sequenced methylotrophic bacteria. Sequences were retreived from Genbank and visualized using CLC Sequence Viewer 5 (www.clcbio.com). Chromosome sequence positions are indicated, as well as the percent identity at the protein level with *Methylobacterium* prototypes (nd: not detectable). Formate tetrahydrofolate ligase/formyl-tetrahydrofolate synthetase (*ftfL*, black); serine hydroxymethyltransferase (*glyA*, pink); serine glyxoylate aminotransferase (*sga*, yellow); hydroxypyruvate reductase (*hprA*, red); glycerate kinase (*gck*, purple); phosphoenolpyruvate carboxylase (*ppc*, orange); malyl-CoA lyase/β-methylmalyl-CoA lyase (*mcl*, dark green); malate thiokinase (*mtkA*/*mtkB*, light green); NAD(P)-dependent methylene-tetrahydromethanopterin/methylene-tetrahydrofolate dehydrogenase (*mtdA*, dark blue); methenyl tetrahydrofolate cyclohydrolase (*fch*, light blue); bifunctional methylene-tetrahydrofolate dehydrogenase/methenyl-tetrahydrofolate cyclohydrolase (*folD*, grey); transcriptional regulator (pale green); other (white); tRNA (black rectangles).

The serine cycle is essential for carbon assimilation in *Methylobacterium* and comprises reactions specific to methylotrophy as well as reactions involved in multicarbon metabolism (Fig. 1, see also [8], [11]). Genes involved in the serine cycle can be ascribed to two categories on the basis of mutational analysis [11]: methylotrophy-specific genes (*glyA*, *sga*, *hpr*, *gck*, *ppc*, *mtkAB* and *mcl*), and genes which are essential under non methylotrophic growth conditions (*eno* and *mdh*). Recent evidence [24], [25] and mutant analyses [23], [70]–[73] suggest that genes for the C1 transfer pathway linked to H_4_F (*mtdA*, *fch* and *ftfL*) are specifically involved in assimilatory metabolism in *Methylobacterium*. Six methylotrophy-specific serine cycle genes, along with *mtdA* and *fch*, belong to gene clusters associated with methylotrophy on the chromosomes of strains AM1 and DM4 (Fig. 4), while the three remaining genes (*glyA*, *gck* and *ftfL*) are not parts of methylotrophy gene clusters and are located elsewhere on the chromosome.

As exemplified here for *M. petroleiphilum* [54], Beta-proteobacterial methylotrophs may also employ the serine cycle for C1 carbon assimilation[54], [74]. As that of the Alpha-proteobacterium *S. pomeroyi*, the genome of *M. petroleiphilum* contains a single gene cluster encoding all required functions of the serine cycle (Fig. 4). In *S. pomeroyi* however, the organisation of this gene cluster is quite different from that of *Methylobacterium*, *Granulibacter bethesdensis* and *M. petroleiphilum* (Fig. 4), and contains tandem genes for two distantly related bifunctional methylene-H_4_F dehydrogenase/methenyl-H_4_F cyclohydrolase (FolD) enzymes instead of the isofunctional *mtdA*/*fch* genes found in the other genomes discussed here. Moreover, the *hpr*, *gck* and *sga* genes inferred from the genomic context display only modest sequence identity with *Methylobacterium* prototypes (Fig. 4), further suggesting that the serine cycle in *S. pomeroyi* belongs to an independent evolutionary lineage.

Extending the analysis to methylotrophic organisms able to grow with methane, the Gamma-proteobacterial methanotroph *M. capsulatus* also harbors serine cycle gene homologs in its genome, including the *mtdA/fch* pair, but few of them are clustered (Fig. 4). However, the gene for one key enzyme of the serine cycle, the methylotrophy-specific phosphoenolpyruvate carboxylase gene, is missing [53], consistent with the extensive biochemical studies demonstrating that the main pathway for C1 assimilation in *Methylococcus capsulatus* is the RuMP pathway.

#### C1 assimilation and the ethylmalonyl-CoA pathway for glyoxylate regeneration

The assimilation of C1 units by the serine cycle requires the regeneration of glyoxylate from acetyl-CoA. It has been a long standing puzzle how strain AM1 achieves this given that it lacks isocitrate lyase activity, the key enzyme of the classical glyoxylate regeneration pathway [8]. Indeed, and the corresponding gene was not detected in the *Methylobacterium* genome. Glyoxylate regeneration via the recently elucidated ethylmalonyl-CoA pathway [12] has now been demonstrated in strain AM1 [13], and the corresponding genes have been identified [11], [12]. The genomes of the other bacteria compared here present a contrasting picture in this respect. The genome of *S. pomeroyi* also contains a complete set of the genes for the ethylmalonyl-CoA pathway (not shown), and as in *M. extorquens*, these genes are not clustered on the chromosome. In *M. petroleiphilum* and *G. bethesdensis*, however, the genes for the key enzymes of the ethylmalonyl-CoA pathway [11] are missing (not shown), but genes thought to encode the isocitrate lyase shunt are present instead [54]. In *M. capsulatus*, neither ethylmalonyl-CoA pathway nor the isocitrate lyase shunt appear to be encoded within the genome [49], consistent with the operation of the RuMP pathway as the predominant pathway for C1 assimilation in *M. capsulatus*.

#### Transcriptional regulation of carbon assimilation in methylotrophic metabolism

The gene of the global serine cycle regulator in *Methylobacterium* (QscR, a LysR-type regulator homologous to CbbR), is essential for methylotrophic growth. It activates transcription of the clustered serine cycle genes as well as of *glyA*, and negatively regulates its own transcription [75] but it is not located in the proximity of known serine cycle genes in the genome. However, the genes of several probable regulators of unknown function are found nearby serine cycle genes in all methylotrophic bacteria including *Methylobacterium* discussed here (Fig. 4).

### Analysis of IS elements uncovers a significant potential for genome plasticity in *Methylobacterium*

**Figure 5:**
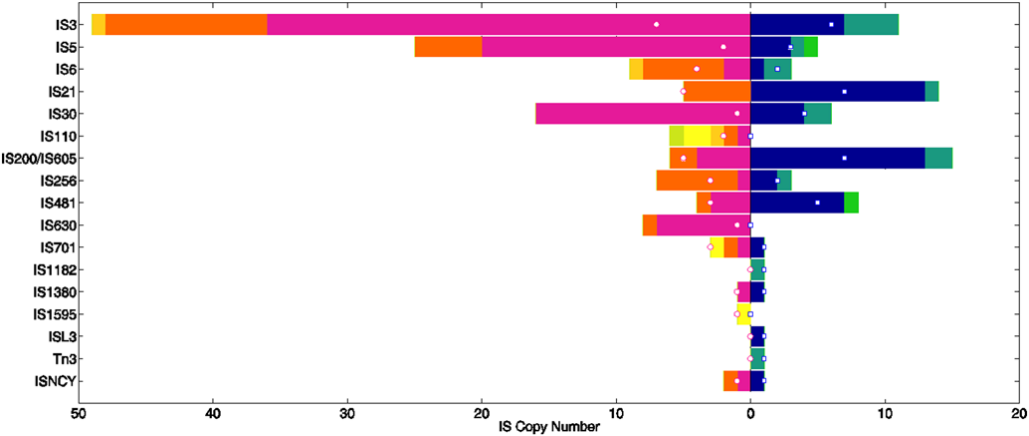
IS family distribution of intact ISs in *M. extorquens* AM1 and DM4. The bar length shows the total intact IS copy number of each IS family in DM4 (right) and AM1 (left) (see Suppl. Table S4). Differently colored regions represent different replicons : blue – DM4 chromosome, cyan – DM4 plasmid p1METDI, green – DM4 plasmid p2METDI, pink – AM1 chromosome, orange – AM1 megaplasmid, dark yellow – AM1 plasmid p1META1, light yellow – AM1 plasmid p2META1, light green – AM1 plasmid p3META1. Open circles and squares represent the numbers of different types of ISs within each family in AM1 and DM4, respectively.

*Methylobacterium* genomes display an IS content comparable to that other microbial genomes [76], but with a clear differential distribution of highly diverse IS elements in AM1 and DM4 (Fig. 5, Suppl. Table S4). In AM1, 39 different IS types (defined by a 95% amino acid identity threshold), belonging to 14 IS families (defined as broad groupings of related elements in ISfinder [77]), were detected, compared to 42 IS types belonging to 14 IS families in DM4. Overall diversity of IS types is higher in DM4, but the total number of IS elements in AM1 is twice as high as in DM4 (Table 1). A total of 71 intact and 23 partial IS elements were detected in strain DM4, representing about 2% of the genome (Table 1). With 9 and 7 copies, respectively, ISMex15 and ISMex17 were the two most abundant IS elements in this strain. In comparison, strain AM1 featured 142 intact and 32 partial IS elements, representing 3.7% of the genome (Table 1). At 37, 16 and 23 intact copies, respectively, ISMex1, ISMex2 and ISMex3 of AM1 (with average pairwise nucleotide differences between different gene copies of only 0.01%, 0.11% and 0.03% respectively), the most abundant IS elements identified, may have undergone recent expansion. In addition, one miniature inverted-repeat transposable element (MITE), MiniMdi3, was detected in both strains (Suppl. Table S4). This element (∼400 bp) is related to ISMdi3 but lacks the transposase gene. Few studies so far have identified the presence of both non-autonomous and autonomous transposable elements in the same bacterial genome (see e.g. Out of a total of 70 IS types identified in this work, only 11 IS types are shared between the two strains (Suppl. Table S4, intact IS). IS*5* and IS*110* are the most abundant shared IS families, each family featuring 5 to 7 different types of IS (Fig. 5). This suggests that substantial IS loss and/or acquisition has occurred during the relatively short period of time since both strains have emerged from a common ancestor.

The distribution of IS element localization within each genome displays clear-cut, non-random features. Plasmids harbor a higher density of IS elements than the chromosomes. Over 20% of the length of the DM4 plasmid p2METDI and of the AM1 plasmids p1META and p2META encode IS elements. Similarly, IS elements comprise about 8% of the length of the AM1 megaplasmid (Table 1), a significantly higher proportion than in the chromosome (*x* ^2^ test, p<0.0001). Moreover, several IS families are significantly over-represented on particular replicons. For example, all 16 copies of ISMex2, an IS element belonging to the IS*481* family that is specific to strain AM1, are found on its chromosome while all 5 copies of the IS elements belonging to the IS*110* family are on the megaplasmid. In contrast, 13 out of the 14 copies of IS elements of this group in DM4 are located on the chromosome. For some IS elements, however, a more homogeneous distribution was noted. For example, the Tn*3* family element ISMex22 unique to strain AM1 is found in one copy per replicon. Transposition immunity was described for this type of IS element [78], suggesting the occurrence of transposition saturation in this case.

The observed non-random IS density across replicons may be due to one or more of three potential causes: (1) biased transposition rates by different IS types across replicons, such as local hopping or plasmid specificity; (2) biased selective effects of transposition events, such as over-representation in regions with high density of genes with little or no selective value, such as plasmids or IS elements themselves; or (3) insufficient time for reaching equilibrium, e.g. for IS elements acquired via recent plasmid-mediated transmission. A second pattern in the distribution of IS locations was noted within each replicon. There is an over-representation of IS elements by 7-fold and 39-fold in chromosomal regions unique to AM1 and DM4, respectively, relative to the regions shared between the two strains (*x* ^2^ test, p<0.0001; also see Fig. 2). These could represent regions with fewer essential genes and therefore relaxed selection against DNA insertions. Alternatively, they could have been IS-rich regions dating back to the common ancestor of these two strains. This would have then led to increased rates of deletion between two co-directional copies of the same IS element, causing such IS-rich regions to be lost more frequently.

#### IS elements linked to methylotrophy

**Figure 6:**
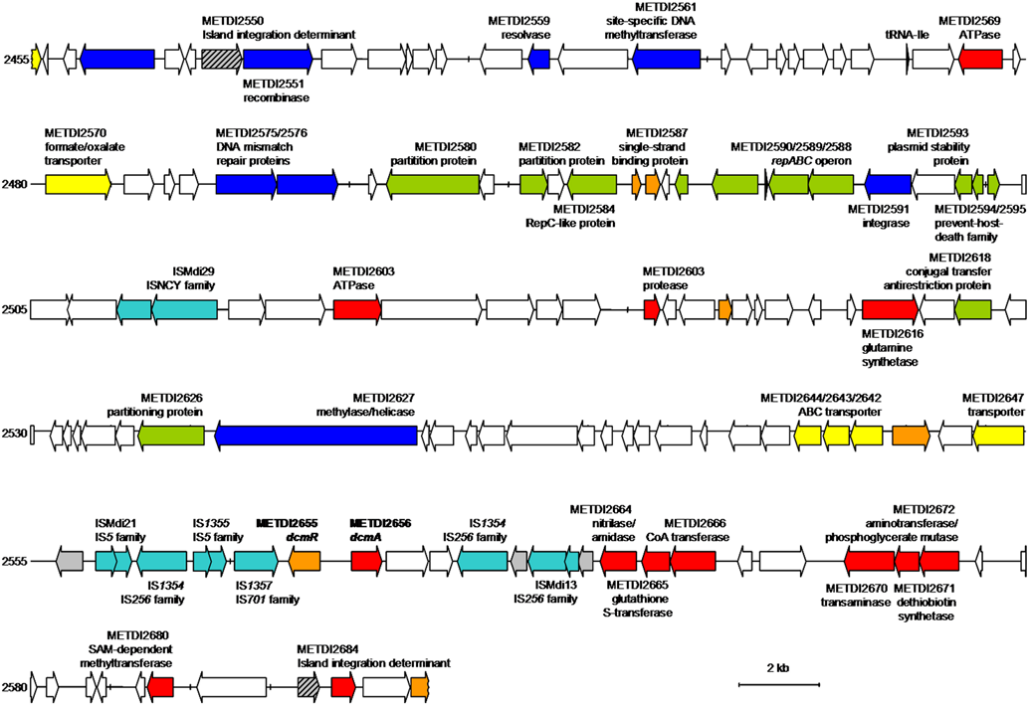
*dcm* region of strain DM4. All functional annotations are putative except for the DCM dehalogenase gene and its upstream regulator (bold). Highlighted are genes for putative enzymes (red), regulators (orange) transporters (yellow), proteins involved in DNA modification (blue), transposases (cyan), proteins involved in plasmid functions (green), and gene fragments (grey), with hypothetical and conserved hypothetical proteins left in white. The interrupted chelatase-likegene (hashed) defined here as “island integration determinant” flanks the 126 kb *dcm* island.

The two strain-specific methylotrophy regions containing *mau* (in AM1) and *dcm* (in DM4) gene clusters (Table 3) are closely associated with IS elements. In strain DM4, genes *dcmR* and *dcmA* are embedded within several overlapping IS elements ([79], Table 3 and see below, Fig. 6). In strain AM1, the *mau* cluster (12 kb) lies between 2 copies of ISMex15 (∼30 kb), as part of a larger (approx. 66 kb) gene cluster unique to this strain (Table 3). This suggests that such methylotrophy-associated gene clusters may be prone to lateral gene transfer and/or deletion. Indeed, it has been shown recently that the presence of the *mau* gene cluster is variable in closely related environmental strains of *Methylotenera*, a betaproteobacterial methylotroph [80]. This phenomenon may be involved in the emergence of new ecotypes of methylotrophs.

### The genomic island for DCM utilization: a new type of mobility determinant?

Unlike methylamine and methanol which are produced naturally in large amounts [2], [81], DCM is produced naturally at low levels only [33], and presumably occurs at significant concentrations in the environment due to industrial production. The *dcm* genomic island unique to strain DM4 with the *dcmA* gene encoding DCM dehalogenase required for growth of *Methylobacterium* with DCM is located on the chromosome (Table 3, Fig. 6), just 20 genes downstream of the large conserved 49 kb methylotrophy gene cluster (Fig. 2, Suppl. Table S1). This 126 kb DNA region, of markedly different GC content (60.5%) from the genome average, was most likely acquired by horizontal transfer. The sequences upstream and downstream of the unique *dcm* region are in complete synteny between the genomes of strains DM4 and AM1. The integration point of the *dcm* region features the 5′-end and 3′-end remains of a “chelatase-like” (COG0606, predicted ATPase with chaperone activity). Although most currently known genomic islands are located at the 3′ end of a tRNA locus, other genes serving as integration sites have been described, such as the *glr* (glutamate racemase) gene of the *Helicobacter pylori* pathogenicity island [82]. Clues on the mode of integration of the *dcm* region within the *Methylobacterium* chromosomal framework were obtained by a more detailed analysis. The first CDS within the *dcm* region encodes a putative recombinase. Arrangements of non-overlapping 5′ and 3′ fragments of such a “chelatase” gene bordering an internal DNA fragment beginning with a recombinase gene are also evident in three other published complete genomes (Table 4). Additional DNA motifs associated with such structures include 5–20 bp direct repeats and palindromic sequences located immediately up- and downstream of the 5′- and 3′-fragments of the disrupted gene, respectively (Table 4). DNA sequences encoding “chelatase” homologs are often apparent pseudogenes, partial sequences, or sequences containing one or several internal stop codons, suggesting that such sequences may have experienced insertion and subsequent excision of DNA fragments. It is tempting to speculate that such sequences represent novel determinants of genome plasticity, and we propose the term “island integration determinant” (*iid*) to describe them.

**Table 4:** **Chelatase**-like Island integration determinant (*iid*) associated with genomic islands in completed microbial genomes.

Characteristic	*M.* strain DM4	*M. extorquens* PA1	*Mesorhizobium loti*	*Nitrobacter hamburgensis* X14
Genome accession number	FP103042	NC_010172	NC_002678	NC_007964
**Disrupted CDS** a				
5′-end fragment	METDI2550 (100)	Mext1904 (99)	Mll4733 (70)	Nham144 (75)
Integration position	329	215	214	214
3′-end fragment	METDI2684 (99)	Mext1923 (98)	Mll4667 (70)	Nham130 (73)
Associated recombinase (Rec)	METDI2551 (+, 100)	Mext1905 (+, 28)	Mlr4668 (+, 28)	Nham143 (+, 28)
Genomic island organization c	DR-DR2-PAL-Rec-[Insert]-PAL/DR2-DR	DR/PAL-Rec-[Insert]-PAL/DR	DR/PAL1-[Insert]-Rec-PAL2/DR	DR-PAL-Rec-[Insert]-PAL/DR
Direct repeat (DR) sequence(s)	GAACC (DR)	TGCTG**ATGA**	**G[C**,G]**CACAAT[C**,G]**T[G**,C]**CT**	CATCA[C,T]TTGCTGA
	AA[T,A]AGA (DR2)			
Palindromic (PAL) sequence(s)	ATTCCCCACCTT>X<AAGGTGGGGAAT	**ATGA**CGTGGCCTATT>X<AATAGGCCACGTCAT	**GCCACAATCT>GCT**A<ACATTGTGGC (PAL1)	GTGATGCTACATTAA>X<TTAATGTAGCATCAC
			GTGCAGTATTAAA>X<TTTAATACGGCACA (PAL2)	
**Associated genomic island**				
Size (kb)	126.4	18.4	50.5	17
%GC d	60.5 (68.1)	69.7 (68.2)	56.2 (62.8)	58.2 (61.7)
Number of CDS	127	18	65	13
Proposed role	DCM degradation	Arsenite resistance	unknown	unknown

a CDS number, % identity at protein level and genomic island integration position relative to the intact island integration determinant CDS META1_1797 of *M. extorquens* AM1.

b CDS number, orientation relative to the upstream island integration determinant and % identity at protein level with the recombinase of strain DM4.

c DR: direct repeat; PAL: palindrome; Rec: recombinase. Slashes indicate sequence overlap, > and < indicate end and begin of mirror palindromic sequences. In *M. loti*, the two palindromic sequence pairs are only separated by three and four bases, respectively. Overlapping segments of direct repeat and palindromic sequences are indicated in bold or underlined (5′-end or 3′-end of the genomic island, respectively). Bases between square brackets indicate alternative bases in the corresponding motif.

d Genome %GC content given in brackets.

The *dcm* region features only few genes that can be associated with confidence with methylotrophic metabolism (Fig. 6). The majority of the genes within this region (74/128, 58%) are hypothetical or conserved hypothetical proteins (compared to the chromosomal average of 41.1% and plasmid average of 43.8% for such proteins, Table 1). Several genes of the *dcm* region are interrupted by IS elements (e.g. a glutathione S-transferase METDI2660/2663), or are present in truncated form (e.g. a DNA helicase METDI2648). Many CDS seem associated with DNA modification, stability and mobility. Moreover, 7 IS elements were identified in this region, with 4 in close proximity to *dcmA* [79]. The structural elements of a *bona fide repABC* plasmid [83], i.e. a canonical *repABC* operon encoding plasmid replication and maintenance function with its counter-transcribed small RNA in divergent orientation upstream of *repC*, and a palindromic 16 nt sequence GTTCTCAGCTGAGAAC fitting the *par* binding site consensus sequence [83] upstream of *repA*, were also found within the *dcm* region. The 8 kb region centered around *repABC* displays extensive synteny with several rhizobial plasmids and with several regions on the chromosome of *Nitrobacter hamburgensis* X14 [84]. This suggests that part or all of the *dcm* region may have once existed as an extrachromosomal element and contributed to the spread of the metabolic capacity to degrade DCM in the environment. Nevertheless, introduction of the *dcmA* gene into strain AM1, with expression of active DCM dehalogenase at high levels, failed to enable growth on DCM [85]. Thus, specific adaptations are required beyond the presence of DCM dehalogenase to enable *Methylobacterium* to grow with this compound [36]. Additional genetic determinants needed for growth with DCM remain to be discovered, and the availability of genomic sequences will facilitate experimental efforts towards identifying them.

### Conclusions

The assembled and complete genome sequences of two strains representing the pink-pigmented facultative methylotrophs of the genus *Methylobacterium* reveal extensive genome-wide homology and gene synteny. Genomic determinants of methylotrophy are almost identical between the two strains, with the exception of the methylamine utilization cluster unique to strain AM1 and of the DCM utilization cluster unique to strain DM4. Still, the two strains differ in genome size and number of replicons, and feature a set of strain-specific genes, mostly of unknown function. The large number and extensive diversity of IS elements in *Methylobacterium* genomes, along with the often clustered organization of genes for utilization of C1 compounds, , suggests that genome rearrangements and horizontal gene transfer most often associated with IS elements, represent key mechanisms of *Methylobacterium* evolution relating to growth-supporting nutrients and environmental conditions.The co-linearity of the two genomes and the absence of substantial large-scale sequence rearrangements are all the more striking in this context, and may indicate that purifying selection sets strong constraints against major alterations of the genome structure in *Methylobacterium*, despite the long laboratory history of the two strains, usually grown with different carbon sources (methanol for strain AM1 and DCM for strain DM4). These two genome sequences thus afford a refined picture of the potential of *Methylobacterium* for physiological flexibility and adaptation to specific environmental constraints within a conserved genomic framework, and provide the basis for renewed, systems level experimental investigations.

## Materials and Methods

### Sequencing, assembly, and validation of the genome of *M. extorquens* AM1

Sequence data were obtained by whole genome shotgun sequencing as previously described [86]. BigDye terminator chemistry and capillary DNA sequencers (model 3700, Applied Biosystems) were used. Randomly picked blunt end-cloned small insert pUC19 vector-based plasmids (average ∼3 kb insert size) were sequenced at both ends using universal forward and reverse sequencing primers, according to standard protocols established at the University of Washington Genome Center. In addition, a large insert fosmid library was constructed from *Sau*3A partial-restricted genomic DNA cloned in *Bam*H1 digested pFOS1 vector. About 1,920 randomly picked fosmid clones were end-sequenced and the data pooled with the small insert shotgun sequence data. Sequence data were assembled and visualized using Phred/Phrap/Consed software (www.phrap.com). The sequence quality and assembly was improved by carrying out several rounds of experiments designed by the Autofinish tool in Consed [87]. Manual finishing was carried out that involved (a) use of specialized sequencing chemistries to sequence difficult regions; (b) PCR amplification and sequencing of specific targeted regions; (c) transposon mutagenesis of over 110 small insert clones followed by sequencing to fix misassembled or difficult to assemble regions; and (d) shotgun sequencing of the 58 targeted fosmid clones to fix long-range misassemblies in the assembled genome. The consensus sequences from transposon mutagenized small insert clones, and the shotgun sequenced fosmid clones were used as backbones in the main genome assembly to resolve misassembled regions. The final strain AM1 genome assembly contained a total of 132942 sequence reads, as well as the backbones from 58 fosmids and over 110 transposon mutagenized small insert clones, and was validated by two independent methods. The gross-scale long-range validity of the genome assembly was established by pulse-field-gel-electrophoresis, with complete agreement between the virtual and experimentally determined fingerprint patterns of the final assembled genome, either by single restriction enzyme digestion with *Pme*I or *Swa*I or by double digestion with a mixture of *Pme*I and *Swa*I restriction enzymes (data not shown). For kb scale validation of the genome assembly, fingerprint data were generated from 1673 of the paired-end-sequenced fosmid clones by digesting with three independent restriction enzymes, *Fsp*I, *Nco*I and *Sph*I. The fosmid paired-end-sequence and experimentally derived fingerprint data were used for assembly validation by comparison with the virtual fingerprint patterns from the assembled genome using the SeqTile software tools developed for this purpose at UWGC [86]. The fosmid paired-end-reads anchored the clone to a unique position in the genome, while the fingerprint data were used to compare experimentally derived fingerprints with the sequence derived virtual patterns. A complete correspondence between the virtual and experimentally derived fingerprint pattern of the genome in the three restriction enzyme domains of *Fsp*I, *Nco*I and *Sph*I was observed, thus validating the genome assembly.

### Genome sequencing, assembly and validation of the genome of strain DM4

The complete sequence of the genome of strain DM4 was obtained using three different libraries. Genomic DNA was fragmented by mechanical shearing, and 3 kb (A) and 10 kb (B) inserts were cloned, respectively, into plasmid vectors pNAV (a pcDNA2.1 (Invitrogen) derivative) and pCNS (a pSU18 derivative). In addition, a large insert BAC library (25 kb inserts, C) was constructed from *Sau*3A partially digested total DNA by cloning into pBeloBAC11. Plasmid DNAs were purified and end-sequenced (79200 (A), 27648 (B), 13056 (C) paired match end-reads, respectively) using dye-terminator chemistry on ABI3730 sequencers. Assembly was realized as described [88] with Phred/Phrap/Consed software package (www.phrap.com). An additional 2170 sequences from selected clones were used in the finishing phase of assembly.

### Genome annotation and bioinformatic analysis

Coding sequences were predicted using the AMIGene (Annotation of Microbial Genomes) software [89] and then submitted to automatic functional annotation using the set of tools listed in [45]. Putative orthology relationships between the two genomes were defined by gene pairs satisfying either the Bidirectional Best Hit criterion [90] or an alignment threshold (at least 40% sequence identity over at least 80% of the length of the smallest protein). These relationships were subsequently used to search for conserved gene clusters (synteny groups) among several bacterial genomes using an algorithm based on an exact graph-theoretical approach [91]. This method allowed for multiple correspondences between genes, detection of paralogy relationships, gene fusions, and chromosomal rearrangements (inversion, insertion/deletion). The ‘gap’ parameter, representing the maximum number of consecutive genes that are not involved in a synteny group, was set to five.

Manual validation of automatic annotations was performed in a relational database (MethylobacScope, https://www.genoscope.cns.fr/agc/mage/wwwpkgdb/Login/log.php?pid=26) using the MaGe web interface [45], which allows graphic visualization of the annotations enhanced by a synchronized representation of synteny groups in other genomes chosen for comparison. Genomes were checked for the presence of genes without homologs in the parent genome using thresholds of 80% sequence identity threshold at the protein level and 80% of the length of the shorter homolog (minLrap 0.8). Chromosomal genes of potentially foreign origin were detected using Alien Hunter [46]. Potential genomic islands were searched for with the RGP (Region of Genomic Plasticity) tool of the Mage web-based interface [45] based on synteny breaks between compared genomes, and then checked the predicted regions manually. Only regions larger than 8 kb are reported here.

IS annotations were done by in-house computational tools (Robinson, Lee, Marx, unpublished) that incorporated IScan [92], followed by manual validation based on ISfinder [77]. IS elements were given names of type “ISMex3”, with “Mex” (for *M. extorquens*) and “Mdi” (for *Methylobacterium* degrading dichloromethane) indicating strains AM1 or DM4, respectively. The same type name was used for both strains for IS elements with >95% identity in protein sequence. An intact copy was defined as a sequence whose length was at least 99% of the length of the longest copy detected, and a partial IS was defined as a >500 bp fragment with >80% DNA identity to an intact copy.

## Supporting Information

Table S1 Methylotrophy genes in M. extorquens AM1 and DM4
(0.07 MB DOC)

Table S2 Methylotrophy enzymes and pathways deduced from complete genomic sequences of methylotrophs
(0.04 MB DOC)

Table S3 Methylotrophic bacteria with published genome sequences included in comparative analyses
(0.04 MB DOC)

Table S4 Characteristics of IS elements in Methylobacterium extorquens
(0.05 MB DOC)
