# Supplementary material for: Methylobacterium Genome Sequences: A Reference Blueprint to Investigate Microbial Metabolism of C1 Compounds from Natural and Industrial Sources
Source: PLoS One. 2009 May 18;4(5):e5584. doi: 10.1371/journal.pone.0005584 (PMC2680597; doi:10.1371/journal.pone.0005584)
Supplement: Table S1 — Methylotrophy genes in M. extorquens AM1 and DM4 (0.07 MB DOC) [file pone.0005584.s001.doc]

**Supplemental Material**

**Supplementary Table S1. Methylotrophy genes in *M. extorquens* AM1 and DM4**

___________________________________________________________________________________Gene Function AM1 DM4 Identity C1

(%) spec.

___________________________________________________________________________________

*pccB* Propionyl-CoA carboxylase, beta subunit META1_0172 METDI0155 99.8 M

*ccr* Crotonyl-CoA carboxylase/reductase META1_0178 METDI0161 100.0 M

*ecm* Ethylmalonyl-CoA mutase META1_0180 METDI0163 99.6 M

*meaB* methylmalonyl-CoA mutase
accessory protein META1_0188 METDI0171 99.7 M

*fdh3A* Cytochrome-linked formate dehydrogenase META1_0303 METDI0454 99.8 M

alpha subunit

*fdh3B* Cytochrome-linked formate dehydrogenase META1_0304 METDI0455 100.0 M

beta subunit

*fdh3C* Cytochrome-linked formate dehydrogenase META1_0305 METDI0456 99.4 R

gamma subunit

*ftfL* Formyl-tetrahydrofolate ligase META1_0329 METDI0483 99.5 M

*qscR* Serine cycle transcriptional regulator META1_0756 METDI1125 99.1 M

*epi* Ethylmalonyl-CoA/

methylmalonyl-CoA epimerase META1_0839 METDI1550 100.0 M

*meaD* ATP:cob(I)alamin adenosyltransferase META1_1433 METDI2206 100.0 M

*mdh* Malate dehydrogenase META1_1537 METDI2311 99.7 E

*sga* Serine glyoxylate aminotransferase META1_1726 METDI2478 100.0 M

*hpr* Hydroxupyruvate reductase META1_1727 METDI2479 99.7 M

*mtdA* Bifunctional methylene-H_4_MPT/ META1_1728 METDI2480 100.0 M

methylene-H_4_F dehydrogenase

*fch* Methenyl-H_4_F cyclohydrolase META1_1729 METDI2481 100.0 M

*mtkA* Malate thiokinase, alpha subunit META1_1730 METDI2482 99.7 M

*mtkB* Malate thiokinase, beta subunit META1_1731 METDI2483 100.0 M

*ppc* PEP carboxylase META1_1732 METDI2484 99.7 M

*mcl* Malyl-CoA lyase/beta-malyl-CoA lyase META1_1733 METDI2485 100.0 M

Hypothetical protein META1_1734 METDI2486 100.0 NA

Putative carboxymethylenebutenolidase META1_1735 METDI2487 96.2

*pcbD* putative pterin-4-Alpha-carbinolamine

dehydratase META1_1736 METDI2488 99.2 NA

ABC type transporter,

substrate-binding subunit META1_1737 METDI2489 100.0

ABC type transporter,

permease subunit META1_1738 METDI2490 98.8

ABC type transporter,

ATP-binding subunit META1_1739 METDI2491 99.6

*xoxF* Homolog of *mxaF* META1_1740 METDI2492 100.0

*xoxG* cytochrome *c* META1_1741 METDI2493 100.0

*xoxJ* Homolog of *mxaJ* META1_1742 METDI2494 98.9

*folK* 2-amino-4-hydroxy-6-hydroxymethyl-7,8-

dihydropterin pyrophosphokinase META1_1743 METDI2495 98.7 NA

*folB* Dihydroneopterin aldolase META1_1744 METDI2496 99.2 NA

*folP* Dihydropteroate synthase META1_1745 METDI2497 99.3 E

Hypothetical protein META1_1746 METDI2498 99.2

Hypothetical protein MEAT1_1747 METDI2499 99.1

*pqqE* PQQ synthesis META1_1748 METDI2500 99.5 M

*pqqC/D* PQQ synthesis META1_1749 METDI2501 98.1 M

*pqqB* PQQ synthesis META1_1750 METDI2502 98.7 M

*pqqA* PQQ synthesis META1_1751 METDI2503 100.0

*mxbM* Transcriptional regulator META1_1752 METDI2504 100.0 M

*mxbD* Sensor kinase META1_1753 METDI2505 99.6 M

Hypothetical protein META1_1754 METDI2506 98.5

*fhcC* Formyltransferase/hydrolase complex

gamma subunit META1_1755 METDI2507 97.7 M

*fhcD* Formyltransferase/hydrolase complex

delta subunit META1_1756 METDI2508 99.7 M

*fhcA* Formyltransferase/hydrolase complex

alpha subunit META1_1757 METDI2509 99.8 M

*fhcB* Formyltransferase/hydrolase complex

beta subunit META1_1758 METDI2510 99.7 M

*mptG* Ribofuranosylaminobenzene

5'-phosphate synthase META1_1760 METDI2512 99.1 M

*mtdB* Methylene-H_4_MPT dehydrogenase META1_1761 METDI2513 100.0 M

*orfY* Unknown META1_1762 METDI2514 95.7 M

*mch* Methenyl-H_4_MPT cyclohydrolase META1_1763 METDI2515 99.7 M

*orf5* H_4_MPT biosynthesis META1_1764 METDI2516 99.3 M

*orf7* Unknown META1_1765 METDI2517 99.3 M

*fae* Formaldehyde activating enzyme META1_1766 METDI2518 100.0 M

*orf17* Unknown META1_1767 METDI2519 96.8 M

*orf9* H_4_MPT biosynthesis META1_1768 METDI2520 97.7 M

Homolog of *mxaE* META1_1770 METDI2522 98.9

Homolog of *mxaD* META1_1771 METDI2523 99.4

Homolog of *mxaD* META1_1772 METDI2524 98.9

*orf19* H_4_MPT biosynthesis META1_1773 METDI2525 98.0 M

*orf20* H_4_MPT biosynthesis META1_1774 METDI2526 98.9 M

*orf21* H_4_MPT biosynthesis META1_1775 METDI2527 95.6 M

*orf22* H_4_MPT biosynthesis META1_1776 METDI2528 97.0 M

*dcmR* Transcriptional repressor not present METDI2655 - M

*dcmA* Dichloromethane dehalogenase not present METDI2656 - M

*fdh4B* Protein associated with expression of

formate dehydogenase 4 META1_2093 METDI2873 98.0 R

*fdh4A* Formate dehydrogenase 4 META1_2094 METDI2874 98.7 R

*msd* Methylsuccinyl-CoA dehydrogenase META1_2223 METDI3005 99.1 M

*folE* GTP cyclohydrolase META1_2264 METDI3046 100.0 NA

*pqqF* PQQ synthesis META1_2330 METDI3110 100.0 M

*pqqG* PQQ synthesis META1_2331 METDI3111 100.0 M

*mcmB* Methylmalonyl-CoA mutase, beta subunit META1_2390 METDI3170 97.5 M

*mauF* Unknown META1_2769 not present - M

*mauB* Methylamine dehydrogenase large subunit META1_2770 not present - M

*mauE* Essential for small subunit maturation META1_2771 not present - M

*mauD* Essential for small subunit maturation META1_2772 not present - M

*mauA* Methylamine dehydrogenase small subunit META1_2773 not present - M

*mauC* Amicyanin META1_2774 not present - M

*mauJ* Unknown META1_2775 not present -

*mauG* Unknown META1_2776 not present - M

*maul* Unknown META1_2777 not present - M

*mauM* Ferredoxin META1_2778 not present -

*mauN* Ferredoxin META1_2779 not present -

*folA* Dihydrofolate reductase META1_2852 METDI3418 97.6 NA

*fumC* Fumarase C META1_2857 METDI3423 99.4 E

*gck* Glycerate kinase META1_2944 METDI3513 98.9 M

*eno* Enolase META1_2984 METDI3551 99.8 E

*pccA* Propionyl-CoA carboxylase, alpha subunit META1_3203 METDI3767 99.3 M

*glyA* Serine hydroxymethyltrasferase META1_3384 METDI3959 100.0 M

*croR* Crotonase META1_3675 METDI4248 100.0 M

*phaR* PHB synthesis regulator/acetyl-CoA flux META1_3699 METDI4271 97.5 M

*phaA* Beta-ketothiolase META1_3700 METDI4272 100.0 M

*phaB* Acetoacetyl-CoA reductase META1_3701 METDI4273 99.6 M

*sdhC* Succinate dehydrogenase, cytochrome

b556 subunit META1_3859 METDI4591 98.5 E

*sdhD* Succinate dehydrogenase,

membrane anchor subunit META1_3860 METDI4592 100.0 E

*sdhA* Succinate dehydrogenase,

flavoprotein subunit META1_3861 METDI4593 99.7 E

*sdhB* Succinate dehydrogenase,

iron-sulfur subunit META1_3863 METDI4595 99.6 E

*mcd* Mesaconyl-CoA hydratase META1_4153 METDI4744 99.1 M

*pabB* para-aminobenzoic acid synthase component META1_4284 METDI4895 98.7 E

*dmrA* Dihydromethanopterin reductase META1_4312 METDI4922 100.0 M

*mxaB* Transcriptional regulator META1_4525 METDI5131 99.6 M

*mxaH* Unknown META1_4526 METDI5132 96.9 NA

*mxaE* Unknown META1_4527 METDI5133 96.5 M

*mxaD* Unknown META1_4528 METDI5134 98.9

*mxaL* Essential for Ca^2+^ insertion into MDH META1_4529 METDI5135 97.9 M

*mxaK* Essential for Ca^2+^ insertion into MDH META1_4530 METDI5136 97.6 M

*mxaC* Essential for Ca^2+^ insertion into MDH META1_4531 METDI5137 98.6 M

*mxaA* Essential for Ca^2+^ insertion into MDH META1_4532 METDI5138 96.8 M

*mxaS* Unknown META1_4533 METDI5139 99.7 M

*mxaR* Unknown META1_4534 METDI5140 99.7 M

*mxaI* Methanol dehydrogenase, small subunit META1_4535 METDI5141 99.0 M

*mxaG* Cytochrome *c*550 META1_4536 METDI5142 99.0 M

*mxaJ* MxaJ, possible chaperone META1_4537 METDI5143 99.0 M

*mxaF* Methanol dehydrogenase, large subunit META1_4538 METDI5145 100.0 M

*fdh2C* Molybdenum-dependent formate

dehydrogenase, gamma subunit META1_4846 METDI5437 100.0 R

*fdh2B* Molybdenum-dependent formate

dehydrogenase, beta subunit META1_4847 METDI5438 98.7 R

*fdh2A* Molybdenum-dependent formate

dehydrogenase, alpha subunit META1_4848 METDI5439 99.7 R

*fdh2D* Molybdenum-dependent formate

dehydrogenase, delta subunit META1_4849 METDI5440 100.0 R

*folC* Dihydrofolate synthase META1_4888 METDI5480 99.3 NA

*mxcQ* Sensor kinase META1_4896 METDI5488 99.0 M

*mxcE* Transcriptional regulator META1_4897 METDI5489 100.0 M

*tenA* Involved in transcriptional regulation META1_4898 METDI5490 97.4

*fdh1B* Tungsten-dependent formate dehydrogenase,

beta subunit META1_5031 METDI5632 99.7 R

*fdh1A* Tungsten-dependent formate dehydrogenase,

alpha subunit META1_5032 METDI5633 99.9 R

*mcmA* Methylmalonyl-CoA mutase, alpha subunit META1_5251 METDI5851 99.7 M

___________________________________________________________________________________

Genes transcribed from the leading strand are highlighted in grey. NA, no mutant available; M, methylotrophy-specific; E, essential for both methylotrophic and non-methylotrophic growth; R, redundant function.
