# Supplementary material for: Methylobacterium Genome Sequences: A Reference Blueprint to Investigate Microbial Metabolism of C1 Compounds from Natural and Industrial Sources
Source: PLoS One. 2009 May 18;4(5):e5584. doi: 10.1371/journal.pone.0005584 (PMC2680597; doi:10.1371/journal.pone.0005584)
Supplement: Table S2 — Methylotrophy enzymes and pathways deduced from complete genomic sequences of methylotrophs (0.04 MB DOC) [file pone.0005584.s002.doc]

**Supplementary Table S2. Methylotrophy enzymes and pathways deduced from complete genomic sequences of methylotrophs**

Pathway/function Organism

_____________________________________________________________________________________________________________

*M. e. G. b. S. p. M. p. M. f. M.* sp. *M. c.* strain V4

_____________________________________________________________________________________________________________

Methane utilization - - - - - - + +

Methanol utilization + + - + + ? + ?

Methylamine utilization + - - - + - - -

H_4_MPT-dependent C1 conversion pathway + + - + + - + -

Formaldehyde oxidation

MtdA/Fch (H_4_MPT-dependent) + + - + - - + -

FolD (H_4_F-dependent) - - + - + + - +

FlhA/FghA (glutathione-dependent) - - + - - - - -

Conversion of formate to CO_2_

Formate dehydrogenase (FDH1) + - + + - - + -

Formate dehydrogenase (FDH2) + + - + + + + +

Formate dehydrogenase (FDH3) + - + + - - - -

Formate dehydrogenase (FDH4) + + - - + - - -

Ribulose monophosphate pathway - - - - + + + -

Calvin-Benson-Bassham pathway - - - + - - + +

Serine cycle + + + + - - partial -

Isocitrate lyase - + - + - - - -

Ethylmalonyl-CoA pathway + - + - - - - -

Tricarboxylic acid cycle + + + + - - + +

_____________________________________________________________________________________________________________
