# Supplementary material for: Methylobacterium Genome Sequences: A Reference Blueprint to Investigate Microbial Metabolism of C1 Compounds from Natural and Industrial Sources
Source: PLoS One. 2009 May 18;4(5):e5584. doi: 10.1371/journal.pone.0005584 (PMC2680597; doi:10.1371/journal.pone.0005584)
Supplement: Table S3 — Methylotrophic bacteria with published genome sequences included in comparative analyses (0.04 MB DOC) [file pone.0005584.s003.doc]

**Supplementary Table S3. Methylotrophic bacteria with published genome sequences included in comparative analyses**

_________________________________________________________________________________________________________________

Organism Phylum Class Genome Growth on Acc. No. Reference

size (Mb) ___________________________

Methanol Methylamine DCM

_________________________________________________________________________________________________________________

*M. extorquens* AM1/DM4 Proteobacteria Alpha 6.9/6.1 + + - This work

*Granulibacter bethesdensis* Proteobacteria Alpha 2.7 + N.D. N.D. NC_008343 [58,59]

*Silicibacter pomeroyi* Proteobacteria Alpha 5.5 N.D. N.D. N.D. NC_003911 [57]

*Methylibium petroleiphilum* Proteobacteria Beta 5.5 + - N.D. NC_008825 [54]

*Methylobacillus flagellatus* Proteobacteria Beta 2.9 + + N.D. NC_007947 [55]

*Methylophilales* sp. HTCC2181 Proteobacteria Beta 1.9 + - N.D. NZ_AAUX00000000 [56]

*Methylococcus capsulatus* Proteobacteria Gamma 3.1 + - N.D. NC_002977 [53]

Strain V4 Verrucomicrobia 2.4 + - N.D. NC_010794 [60]

_________________________________________________________________________________________________________________

N.D., not detected
