# Supplementary material for: Methylobacterium Genome Sequences: A Reference Blueprint to Investigate Microbial Metabolism of C1 Compounds from Natural and Industrial Sources
Source: PLoS One. 2009 May 18;4(5):e5584. doi: 10.1371/journal.pone.0005584 (PMC2680597; doi:10.1371/journal.pone.0005584)
Supplement: Table S4 — Characteristics of IS elements in Methylobacterium extorquens (0.05 MB DOC) [file pone.0005584.s004.doc]

**Supplementary Table S4. Characteristics of IS elements in *Methylobacterium extorquens***

________________________________________________________________________________

IS type Length IS family DM4 AM1

(bp) ________________ _________________

Intact Partial Intact Partial

________________________________________________________________________________

ISMex1 1200 IS*3* 0 0 37 1

ISMex2 1041 IS*481* 0 0 16 0

ISMex3 1399 IS*256* 2 0 23 0

ISMex4 1615 IS*1380* 0 0 8 0

ISMex5 1283 IS*3* 0 4 4 5

ISMex6 1535 ISNCY 0 0 2 0

ISMex7 1285 IS*3* 0 0 1 0

ISMex8 2597 IS*21* 0 0 3 0

ISMex9 1384 IS*110* 0 0 1 1

ISMex10 1762 ISL*3* 2 0 4 0

ISMex11 1227 IS*3* 3 1 2 3

ISMex12 1659 IS*110* 0 1 1 1

ISMex13 2359 IS*21* 0 0 4 0

ISMex14 1381 IS*256* 0 0 2 0

ISMex15 836 IS*5* 9 1 2 1

ISMex16 1511 IS*3* 1 0 3 2

ISMex17 1132 IS*110* 7 1 1 0

ISMex18 1173 IS*630* 1 0 1 2

ISMex19 851 IS*5* 0 0 1 0

ISMex20 2321 IS*200*/IS*605* 0 0 1 0

ISMex21 1046 IS*630* 2 0 2 0

ISMex22 3864 Tn*3* 0 0 5 1

ISMex23 1548 IS*200*/IS*605* 0 0 1 0

ISMex24 1678 ISL*3* 0 0 2 0

ISMex25 887 IS*6* 0 0 1 1

ISMex26 1872 ISL*3* 0 0 1 2

ISMex27 2493 IS*21* 0 0 1 0

ISMex28 1523 IS*5* 0 0 1 0

ISMex29 1334 IS*110* 1 0 1 0

ISMex30 1047 IS*630* 1 1 1 0

ISMex31 1081 IS*110* 1 0 1 0

ISMex32 873 IS*5* 0 0 1 0

ISMex33 1318 IS*3* 0 1 1 0

ISMex34 636 IS*200*/IS*605* 0 0 1 0

ISMex35 1560 IS*5* 0 0 1 0

ISMex36 1475 IS*1182* 0 0 1 0

ISMex37 1510 IS*3* 0 0 1 2

ISMex38 3843 Tn*3* 0 0 1 0

ISMex39 2440 IS*21* 0 0 1 0

IS1354 1431 IS*256* 2 0 0 0

IS1355 970 IS*5* 1 0 0 0

IS1357 1229^*^ IS*701* 0 1 0 0

ISMdi1 1064 IS*630* 3 1 0 1

ISMdi2 1691 ISL*3* 1 0 0 1

ISMdi3 1246 IS*3* 4 4 0 4

ISMdi4 1063 IS*481* 2 3 0 0

ISMdi5 1279 IS*3* 1 1 0 1

ISMdi6 1476 IS*1182* 1 0 0 0

ISMdi7 2568 IS*21* 2 0 0 1

ISMdi8 1006 IS*481* 2 0 0 1

ISMdi9 1625 IS*110* 1 0 0 0

ISMdi10 1437 IS*110* 1 0 0 0

ISMdi11 1894 IS*481* 1 0 0 0

ISMdi12 1361 IS*110* 1 0 0 0

ISMdi13 1416 IS*256* 1 0 0 0

ISMdi14 1341 IS*110* 2 0 0 0

ISMdi15 1889 IS*200*/IS*605* 1 0 0 0

ISMdi16 1271 IS*3* 1 0 0 0

ISMdi17 890 IS*5* 1 0 0 0

ISMdi18 999 IS*1595* 1 1 0 0

ISMdi19 1099 IS*3* 1 0 0 0

ISMdi20 1062 IS*630* 1 1 0 0

ISMdi21 942 IS*5* 1 0 0 0

ISMdi22 724 IS*5* 1 0 0 0

ISMdi23 1065 IS*481* 1 0 0 0

ISMdi24 855 IS*5* 1 0 0 0

ISMdi25 1601 IS*30* 1 0 0 0

ISMdi26 867 IS*5* 1 1 0 1

ISMdi27 2464 IS*21* 1 0 0 0

ISMdi28 1410 IS*701* 1 0 0 0

ISMdi29 1939 IS*NCY* 1 0 0 0

MiniMdi3 416^‡^ 8 0 4 0

________________________________________________________________________________________________________________

^*^ fragment length

^‡^ Maximum length. Actual lengths vary from 382 to 416.
